# Explaining success in Bayesian reasoning with the adaptive toolbox and expertise

**DOI:** 10.3758/s13423-026-02986-5

**Published:** 2026-08-17

**Authors:** Hannah Fenwick, Guillermo Campitelli, Matthew B. Thompson

**Affiliations:** 1 School of Psychology, Murdoch University, Murdoch, Australia; 2 Centre for Biosecurity and One Health, Harry Butler Institute, Murdoch University, Murdoch, Australia

**Keywords:** Bayesian reasoning, Ecological rationality, Nested sets account, Expertise, Adaptive Toolbox, Human rationality

## Abstract

Research on Bayesian reasoning has been shaped by two productive traditions: ecological rationality, which explains why natural frequencies facilitate inference, and nested sets accounts, which show how transparent set relations support analytic reasoning. Together, these approaches have generated a rich empirical literature on representational facilitation. Yet a broader pattern has emerged: Bayesian reasoning accuracy improves with training and visualisation, varies systematically with numeracy and domain knowledge, and can be high among mathematically trained reasoners even in probability formats. These findings suggest that Bayesian competence is not reducible to a privileged input format but reflects the acquisition and deployment of multiple inferential tools. Building on prior integrative contributions, we propose that Gigerenzer’s adaptive toolbox, combined with principles from expertise research, provides a productive theoretical framework and research agenda. From this perspective, natural frequencies and nested set representations function as scaffolds that make particular tools easier for novices to access, rather than as the sole route to success. An expertise-based interpretation helps explain how inferential tools are learned, automatised, and selected through experience, why tool use varies across individuals and tasks, and how accurate Bayesian reasoning can be achieved across representational formats. We further argue that expert–novice comparisons and process-tracing methods provide a powerful approach to identifying the tools that support probabilistic inference and to guiding the design of more generalisable training interventions. This framework links representational facilitation to learning and strategy selection, shifting the focus of Bayesian reasoning research from which formats work best to how people become capable of Bayesian reasoning at all.

Bayesian reasoning, the capacity to update beliefs in light of new evidence, is widely regarded as an important component of rational decision-making under uncertainty and risk^1^. From medical diagnosis to legal judgment, the ability to integrate priors with new information shapes critical real-world outcomes. Yet decades of research show that most people perform poorly on Bayesian inference tasks, particularly when information is presented in standard probability formats, with success rates estimated as low as 4%. Such figures underscore widespread failure, but they also highlight that success, however infrequent, does occur.

In this review, we start from those successful cases. The dominant focus in Bayesian reasoning research has been on demonstrating performance improvements when information is presented in simplified natural frequency formats compared to abstract probability formats—a goal that has produced substantial advances in our understanding of representational facilitation. However, even with frequency formats, accuracy remains moderate, typically ranging from 20 to 50% depending on task features (McDowell & Jacobs, 2017), and considerable variability across individuals and contexts remains unexplained. Moreover, systematic improvements occur *within* probability formats through training, visual aids, and individual differences in numeracy and domain expertise—yet these receive less theoretical attention because they fall outside the natural frequency-versus-conditional probability comparison that has structured much of the field’s empirical work.

Despite low average performance, several robust findings indicate that Bayesian reasoning competence is systematically achievable. Training studies demonstrate that it can be learned and retained in both natural frequency and probability formats (e.g., Kim et al., 2024; Sedlmeier & Gigerenzer, 2001). Visual aids reliably improve performance by clarifying reference classes and set relations (see Cui et al., 2023, for a review of visual aids used). Individual difference measures for numeracy, cognitive reflection, visual spatial ability and working memory predict accuracy (e.g., Brase, 2021b; Sirota & Juanchich, 2011). Most critically, a consistent minority of participants solve probability-format problems without scaffolding, and recent evidence suggests that mathematically trained experts can achieve high accuracy across challenging probability formats (Fenwick et al., manuscript in preparation).

Prior work has established that Bayesian performance depends on factors beyond representational format, and some contributions have proposed frameworks integrating these factors, including accounts centred on verbal comprehension, relational reasoning, and task structure (Johnson & Tubau, 2015, 2017; Sirota et al., 2015b, 2024; Tubau, 2022; Tubau et al., 2023). We build on this literature but ask a broader question: not why particular formats reduce error, but what inferential tools support Bayesian reasoning and how they are acquired. We integrate Gigerenzer’s (2001) adaptive toolbox, developed to explain judgment and decision-making more broadly, with expertise research from cognitive science, and propose this integration as an initial theoretical framework and empirical research agenda for pursuing this question. Expertise research offers well-developed theoretical principles for understanding how the mind acquires, organizes, and utilises knowledge to achieve skilled performance across diverse cognitive domains including chess (Campitelli et al., 2014; Chase & Simon, 1973; De Groot, 1978), physics problem solving (Larkin et al., 1980), radiology screening (Myles-Worsley et al., 1988), and forensic identification such as fingerprint matching (Thompson & Tangen, 2014). Although the adaptive toolbox has been invoked in some instances of Bayesian reasoning research, its application has focused primarily on natural frequency-based representations as the privileged input for a narrow set of cognitive tools (e.g., Hoffrage et al., 2015). In contrast, Gigerenzer’s broader adaptive toolbox framework allows that effective reasoning could depend on a greater repertoire of tools; frequency-based, set-based, causal, and probabilistic, that are selected and deployed based on task demands and prior knowledge. An expertise perspective could help explain *how* individuals acquire this fuller repertoire of tools, *why* tool selection varies systematically across individuals and contexts, and *when* natural frequency-based facilitation emerges as one strategy among many rather than a privileged default. We propose that examining how mathematical and statistical experts reason successfully with probability formats offers a promising avenue for testing this framework: expert-novice comparisons could reveal the cognitive processes, representational strategies, and reasoning shortcuts experts use to solve problems that stymie novices, providing empirically grounded guidance for supporting novice reasoning.

The proposal presented in this article aligns with the calls for theoretical integration in the field. Brase and Hill (2015) argue that progress “depends on continued research that honestly, vigorously, and consistently engages across these different theoretical accounts rather than staying ‘siloed’ within one particular perspective” (p. 1). McNair (2015) notes that current dominant approaches leave major variability in facilitation effects unexplained and urges research to move beyond the ecological-versus-nested sets divide to understand who succeeds and why to advance the field in both method and theory. Likewise, Mandel (2014) highlights the need for richer accounts of “what it is to ‘be Bayesian’,” emphasising that existing methods reveal little about the cognitive processes behind successful Bayesian reasoning.

Rather than adjudicating between existing theories, we treat them as complementary components that illuminate different cognitive tools people bring to Bayesian problems. Our aim is to place successful performance—not failure—at the centre of integration. As Gigerenzer (2008) states, “A major goal for all theorists must be to integrate what exists rather than to neglect or denigrate the rest of psychology. Connecting theories conceptually exposes our mutual blind spots and can lead to new and bold insights” (p. 27). This call to connect theories and expose mutual blind spots is precisely what motivates our review. Section 1 introduces Bayesian reasoning and the conditional probability versus natural frequency distinction, situates this research within broader rationality debates, and reviews the dominant theoretical accounts of ecological rationality and nested sets. Section 2 examines evidence from training, visual aids, individual differences, and task manipulations, alongside additional theoretical accounts, revealing systematic patterns of success that extend beyond format-based facilitation. Section 3 returns to Gigerenzer’s broader adaptive toolbox framework, demonstrating how it accommodates diverse cognitive tools and establishing the conceptual foundation for an expertise-based approach. Section 4 develops the background and rationale for an expertise-based approach to integration, explaining how expertise research provides theoretical tools to address patterns current accounts leave unexplained, and proposing expert-novice comparisons as a promising direction for future investigation.

## Section 1: Bayesian reasoning

One of the most empirically tested methods for improving performance on Bayesian reasoning tasks is to manipulate the representational format using *natural frequencies*. Natural frequencies are whole-number counts that represent how often specific events occur (e.g., “10 out of 100”), preserving the underlying sampling structure and making part–whole relations transparent (see McDowell & Jacobs, 2017). Gigerenzer and Hoffrage’s (1995) seminal studies demonstrated that converting standard percentage-based problems into natural frequencies substantially increases accuracy and initiated decades of research into understanding the mechanisms underlying this facilitation effect. To understand why many people struggle with conditional probabilities but perform better with natural frequencies, it is useful to recall the structure of Bayes’ theorem (Bayes, 1763)—the normative formula for updating beliefs given new information:

### Bayes theorem

1 $$P\left(H|D\right)=\frac{P(D|H)\times P(H)}{P(D)}$$

Here, P denotes Probability, H denotes Hypothesis, and D denotes Data. The posterior probability, P(H∣D), reflects the probability that a hypothesis holds given the observed data. Prior to observing the data, P(H) represents the prior probability, the initial probability assigned to the hypothesis, often referred to as the base rate. The likelihood, P(D∣H), captures the probability of observing the particular data under the assumption that the hypothesis is true; alternative terms for this component include hit rate and sensitivity. The marginal likelihood, P(D), refers to the overall probability of the observed data. Because P(D) is rarely provided explicitly in textbook problems, it must be intuitively calculated through the expanded formulation for the hypothesis being both true and false:

2 $$P\left(H|D\right)=\frac{P(D|H)\times P(H)}{P\left(D|H\right)\times P\left(H\right)+P(D|\neg H)\times P(\neg H)}$$

**Fig. 1:**
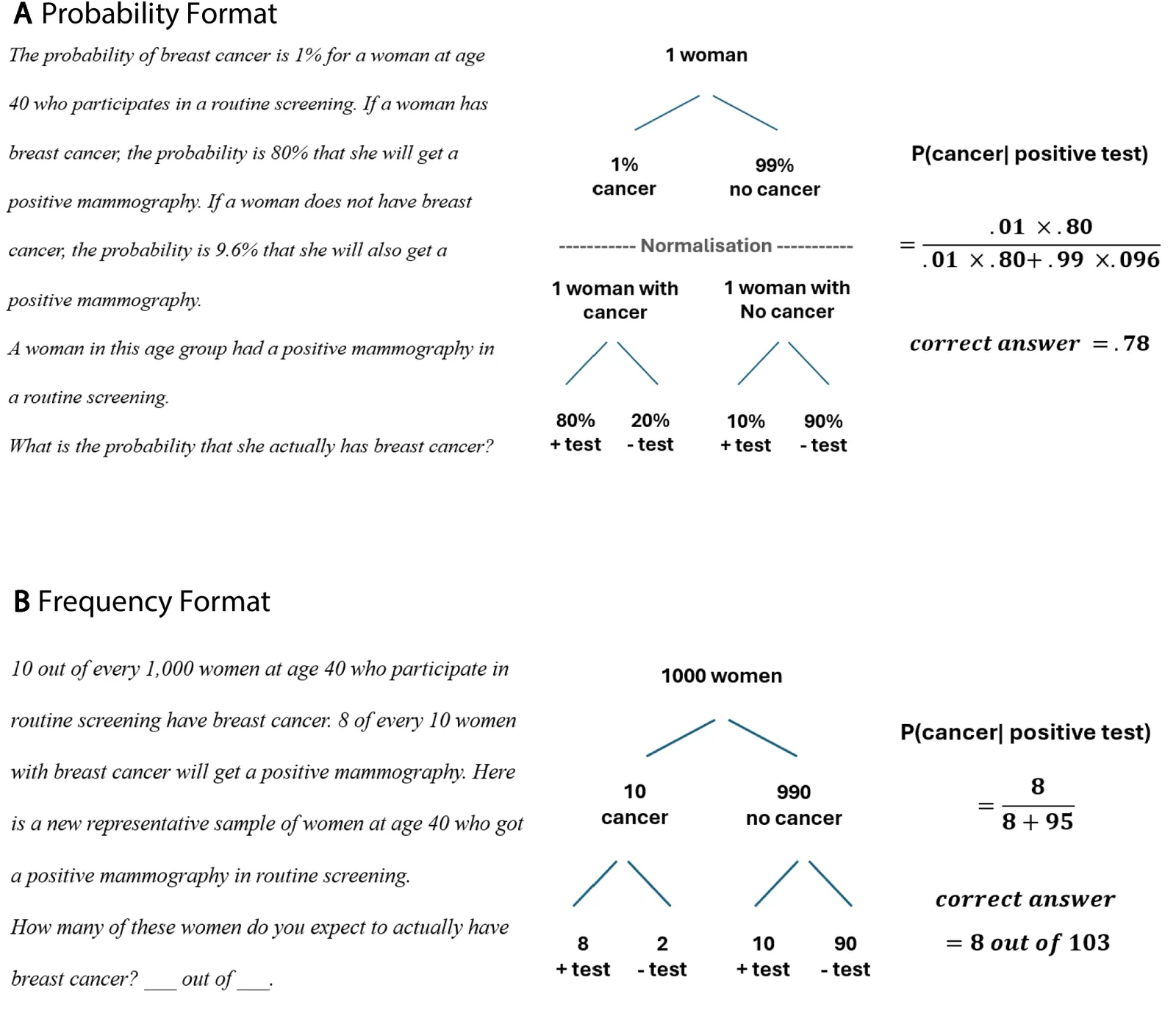
Bayesian reasoning mammography problem in conditional probability and natural frequency formats. **A** Probability format and **B** frequency format of textbook Bayesian task the mammography problem showing the structure of the problem and the formula required to solve each version—figure is inspired by structure presented in Gigerenzer and Hoffrage (2007) and Mammography problem as worded in Gigerenzer and Hoffrage (1995)

Figure 1A illustrates how this computation is instantiated in a classic conditional probability-format mammography problem. Figure 1B presents the same information in natural frequencies, where the Bayesian solution reduces to comparing counts in a simplified structure:

Standard Frequency Format Equation [ *f* (.) indicates frequencies]:

3 $$\mathrm{P}\left(\left.\mathrm{H}\right|\mathrm{D}\right)=\frac{ f (D\cap H)}{f (D\cap H) + f (D\cap \neg H)}$$

Meta-analyses estimate that performance improves from approximately 4% in conditional probability formats to around 24% in natural frequency formats (McDowell & Jacobs, 2017). This robust facilitation effect motivated the development of two major theoretical frameworks in Bayesian reasoning: the ecological rationality account (Gigerenzer & Hoffrage, 1995) and the nested sets account (see Johnson-Laird et al., 1999; Sloman et al., 2003). Both approaches focus primarily on explaining why natural frequency formats improve performance relative to conditional probability formats. However, this focus forms part of a broader debate about human rationality—whether systematic errors in probabilistic reasoning reveal fundamental cognitive biases and limitations, as argued in the heuristics-and-biases tradition (Kahneman & Tversky, 1973), or whether such errors arise from mismatches between our cognitive repertoire and the structure of experimental tasks, as emphasized by ecological rationality and fast-and-frugal heuristics approaches (Gigerenzer, 1996). To understand how ecological rationality and nested sets accounts emerged it is necessary to situate these accounts within the historical development of rationality theories in psychology.

#### Section 1.1: Emergence of theoretical accounts of human rationality in psychology

The question of whether humans are fundamentally rational or prone to systematic error has shaped psychological theory for more than a century. Early perspectives, influenced by Enlightenment ideals and classical economic theory, assumed that people reasoned according to normative principles of logic and probability (Friedman & Savage, 1948; Laplace, 1774). In psychology, Phillips and Edwards (1966) proposed that individuals function as *intuitive statisticians*, producing judgments broadly consistent with Bayesian principles, though often conservative in updating.

The view of humans as perfect rational agents was challenged by Herbert Simon (1955, 1956), who introduced *bounded rationality.* Simon argued that people aim to be rational but operate under constraints of limited knowledge, time, and computational capacity. Rather than seeking optimal solutions, individuals adopt “satisficing” behaviour, relying on heuristics—experience-based strategies that yield “good-enough” solutions within these constraints (Simon, 1986).

Kahneman and Tversky’s (1973, 1996) *heuristics-and-biases* programme shifted the emphasis to an inherent fallibility in rationality by demonstrating that people rely on mental shortcuts that, while often efficient, produce predictable and systematic errors when applied to problems requiring normative probability reasoning. These findings were interpreted as evidence that human judgment is fundamentally biased and unreliable under uncertainty (Tversky & Kahneman, 1974).

Gigerenzer later reconceptualised heuristics as *ecologically rational* rather than sources of error and bias. Drawing on Simon’s (1956) framework and Brunswik’s (1955) functionalism, he proposed that heuristics are well adapted to the informational structures of real environments and should be evaluated by ecological fit rather than conformity to formal norms (Gigerenzer & Goldstein, 1996). This view developed into the *ecological rationality account* (i.e., Goldstein & Gigerenzer, 2002), which treats successful reasoning as emerging from how well cognitive processes exploit the structure of the environment.

These theoretical shifts, from rational agents to bounded rationality, to biased judgment, and finally to ecologically adaptive cognition, form the backdrop against which current accounts of Bayesian reasoning were developed. The ecological rationality account and the nested sets account represent the two dominant approaches for explaining why Bayesian reasoning improves under certain representational conditions, particularly with natural frequencies.

#### Section 1.2: Ecological rationality account in Bayesian reasoning

According to accounts of ecological rationality natural frequencies make Bayesian inference simpler by substantially reducing the computational complexity of the task. Natural frequencies are ecologically valid because they mirror real-world sampling processes that suggest humans experience events as counts rather than abstract probabilities (Hoffrage et al., 2002). As a result, frequency formats align with how memory encodes statistical information and supports the efficient use of simple strategies (Gigerenzer, 1996). Gigerenzer and Hoffrage (1995) explain, “Evolutionary theory asserts that the design of the mind and its environment evolve in tandem. . . . We assume that as humans evolved, the ‘natural’ format was frequencies as actually experienced in a series of events, rather than probabilities or percentages” (p. 686). Thus, the mind is proposed to be better tuned to representations encountered in our evolutionary history rather than abstract probabilities which are a modern invention. Ecological rationality does not claim that correct Bayesian responding is impossible in probability formats; rather, its explanatory emphasis has been on why frequency-like inputs reduce computational demands for novices.

Crucially, natural frequencies enable fast-and-frugal strategies to approximate Bayesian reasoning without formal computation of conditional probabilities. When presented in natural frequencies, Bayesian inference becomes a matter of simple counting operations, reducing the number of mental steps required and exposing the information structure that heuristics can exploit (Gigerenzer, 1996; Gigerenzer & Goldstein, 1996). Natural frequencies, therefore, function as ecological input that allows a simple strategy to approximate optimal inference. Importantly, Gigerenzer and Hoffrage (1995) did not argue that humans lack Bayesian algorithms, but that algorithms are attuned to particular representational formats. As they put it, “our argument centres on the intimate relationship between a cognitive algorithm and an information format” (p. 685). Their calculator analogy illustrates the point: a calculator designed for Arabic numerals (1, 2, 3) will fail with Roman numerals (I, II, III) not because it lacks computational ability, but because the input format mismatches its design. Likewise, people may appear to reason poorly when presented with probability formats that obscure the relevant natural sampling structure (see also Kleiter, 1994).

In Bayesian reasoning, probability formats require multiple fractional computations and explicit integration of base rates, whereas natural frequency formats reduce the task to tracking two absolute frequencies (hits and false alarms). As Gigerenzer and Hoffrage (1995) noted, “neglect of base rates is perfectly rational in natural sampling” (p. 687) because the base rate is embedded within the natural frequency structure itself. Accordingly, the facilitation effect is attributed to ecological fit rather than computational deficiency.

Parallel to ecological rationality accounts, evolutionary psychologists extended this argument by proposing evolved cognitive modules specialised for processing frequencies. Cosmides and Tooby (1996) articulated the *frequency hypothesis*: “the hypothesis that some of our inductive reasoning mechanisms do embody aspects of a calculus of probability, but they are designed to take frequency information as input and produce frequencies as output” (p. 3). In this view, facilitation effects arise not merely from representational fit but from the activation of evolved inductive mechanisms. The *individuation hypothesis* provides an explanation for this evolutionary interpretation, by showing that Bayesian reasoning improves when problems involve individuated, countable objects that activate these specialised evolved modules (Brase et al., 1998).

Brase (e.g., 2002, 2021a) has been a long-standing and influential proponent of ecological rationality in the Bayesian reasoning literature, often overlapping the evolutionary frequency hypothesis and ecological rationality approaches. As Brase (2021b) puts it, ” the human mind should be expected to be better at tasks (including Bayesian reasoning) when they are presented in ways that are more consistent with the natural and evolutionary ecology of the real-world environment” (p. 236). In this way, Brase positions Bayesian facilitation not merely as an artifact of representational format, but as evidence that evolved reasoning mechanisms are tuned to ecologically valid structures; thus, embedding the evolutionary frequency hypothesis within Gigerenzer’s ecological rationality account.^2^

#### Section 1.3: Nested sets account

The Nested sets account (e.g., Barbey & Sloman, 2007; Johnson-Laird et al., 1999; Sirota et al., 2015a; Sloman et al., 2003) has often been positioned in the Bayesian reasoning literature as a main theoretical alternative to ecological rationality, even though later exchanges have highlighted substantial overlap between the two accounts, particularly regarding set–subset structure (see Section 1.4). Originating in the heuristics-and-biases tradition, the nested sets perspective argues that facilitation occurs not because humans possess an evolved frequency-processing module but because certain representational formats make the set–subset relationships in Bayesian problems more transparent. In this view, natural frequencies improve performance insofar as they clarify hierarchical relations among categories, but they are not uniquely privileged. For example, in the mammography problem, the statement “1 out of 1,000 women have cancer” makes explicit the subset relation between affected and unaffected individuals. The subsequent information “8 of every 10 women with cancer test positive” clarifies that the 8 is nested within the 10, which itself is nested within the 1,000. According to nested sets proponents, it is this explicit hierarchical structure, not the frequency format per se, that drives facilitation (see Girotto & Gonzalez, 2001).

Two explanatory approaches have been used within nested sets accounts to explain these effects. First, dual-process theories (Evans, 2003; Kahneman, 2011) propose that Bayesian reasoning depends on whether problems cue intuitive or deliberative processing. Standard probability formats tend to elicit fast, heuristic (System 1) responses that are prone to base-rate neglect. Natural frequency formats, however, highlight the nested structure of the data—showing how subsets are embedded within larger sets (e.g., cancer cases nested within all positive tests). This explicit representation of inclusion relations can cue analytic (System 2) reasoning, enabling the application of set-based rules such as computing ratios of joint to total cases (Barbey & Sloman, 2007; Lesage et al., 2013). When probability formats obscure these relations by normalising values relative to the population base, analytic reasoning is less likely to be engaged, leaving heuristic responses to dominate, often resulting in errors.

Second, the mental models account proposes that individuals solve Bayesian problems by constructing concrete set-based representations of the situation. Probability formats obscure these mental models, whereas frequency formats and visual displays (e.g., Venn diagrams, tree diagrams) make part–whole relationships more explicit and therefore easier to represent mentally (Johnson-Laird, 1994; Johnson-Laird et al., 1999, 2015). From this view, clearer set structures allow reasoners to interpret the problem more effectively, facilitating correct inference.

The nested sets account therefore makes predictions distinct from ecological rationality. Any representation that makes set relationships salient, not only natural frequencies, should improve Bayesian reasoning. Performance should also vary systematically with individual differences in analytic ability, as recognising and manipulating set structures draws on general cognitive resources (Lesage et al., 2013). Finally, from a nested sets perspective, visual aids that clearly depict set inclusion should be particularly effective in supporting Bayesian reasoning (Sirota et al., 2014).

#### Section 1.4: Theoretical exchanges

The contrast between ecological rationality and the nested sets account generated an extended theoretical dialogue that has shaped Bayesian reasoning research for more than two decades. Several authors argued that the natural frequency advantage reflected reduced computational demands and clearer representation of set–subset relations rather than specialised frequency-processing mechanisms. For example, Macchi and Mosconi (1998) emphasised the role of partitive structures, Lewis and Keren (1999) highlighted the distinction between conditional and joint representations, and Mellers and McGraw (1999), Johnson-Laird et al. (1999), Evans et al. (2000), and Girotto and Gonzalez (2001) proposed that facilitation could also emerge in nonfrequency formats when relational structure was made explicit. These findings converged on the idea that transparent set relations, rather than frequency format alone, play an important role in Bayesian reasoning performance.

Ecological rationality proponents responded that many critiques conflated natural frequencies with frequencies more generally. Gigerenzer and Hoffrage (1999, 2007) argued that natural frequencies possess distinctive structural properties because they preserve information generated through natural sampling and explicitly encode base-rate information (see also Hoffrage et al., 2002). They further contended that several mechanisms proposed by nested sets researchers, including set–subset relations and computational simplification, were already in the original Gigerenzer and Hoffrage (1995) ecological rationality framework. Although these exchanges substantially refined theoretical understanding of representational facilitation, both traditions largely focused on explaining performance differences between formats. As a result, influential findings such as training effects, individual differences, and successful reasoning across diverse representations remained secondary theoretical targets. Section 2 therefore examines what existing theories explain about successful Bayesian reasoning and where additional explanatory mechanisms may be required.

## Section 2: What current theories explain about successful Bayesian reasoning

This section focuses on evidence from training studies, visual aids, individual differences, and task manipulations to characterise the broader pattern of successful Bayesian reasoning that extends beyond format-based facilitation.

### Section 2.1: Training studies

Training studies provide some of the strongest evidence for success in Bayesian reasoning. Across interventions, instruction, practice, and representational scaffolding reliably increase accuracy in both natural-frequency and probability formats.

Sedlmeier and Gigerenzer (2001) compared rule training (inserting probabilities into Bayes’ theorem) with representation training (constructing natural-frequency grids or trees). Baseline accuracy was near zero, but post-training accuracy rose to about 60% for rule training and 75–90% for frequency representation training, with both methods transferring to novel problems. Retention diverged: rule-trained participants fell to around 20% after 5 weeks, whereas frequency-trained participants maintained high accuracy. Importantly, incentives dramatically altered this trajectory: When rule-trained participants were rewarded, they sustained approximately 70% accuracy; interviews indicated deliberate review practice had been done by some participants in order to receive the incentive bonus. Sedlmeier and Gigerenzer (2001) explained the superiority of natural frequency training by arguing that “the mind has evolved and learned analogous preferences for particular formats . . . natural frequencies correspond to the format of information humans have encountered throughout most of their evolutionary development” (pp. 381–382), and that natural-frequency representations “do part of the cognitive work” (p. 381). At the same time, they acknowledged that “nobody denies that students can, in principle, learn to apply Bayes’ rule successfully (otherwise, there would be no experts in statistics)” (p. 394), but they did not develop a corresponding account of how probability-based strategies are learned, retained, and generalised. The findings, therefore, indicate that durable success in probability formats is achievable through training and through motivated practice—yet this pathway lies outside the explanatory scope of ecological rationality as presented by the authors.

Similar patterns appeared in Kurzenhäuser and Hoffrage (2002) who tested a 1-hour classroom tutorial for medical students, comparing representation training (translating probabilities into natural frequencies using frequency trees) with rule training (applying Bayes’ formula). Baseline performance was near zero (0.9% and 3%, respectively). Two months later, 47% of students in the frequency group produced correct Bayesian solutions, compared with 16% in the rule group. They attributed the advantage of natural frequencies to natural sampling, arguing that “the necessary mental algorithms are computationally easier to perform with natural frequencies than with probabilities . . . [because] natural frequencies implicitly carry information on base rates in a population” (p. 517). This framing explains relative superiority but does not illuminate the representational or cognitive mechanisms supporting success in the rule-training group. Across both studies, gains in probability formats indicate that instruction can cultivate Bayesian competence even under representational conditions considered challenging by both ecological rationality and nested sets accounts.

Further evidence comes from Sirota et al. (2015b), who examined whether training effects reflect a format-representation shift (conditional probabilities translated into natural frequencies; ecological rationality prediction) or a problem-representation transfer (learning a nested sets schema; nested sets prediction). Students trained with either chance/risk wording or frequency wording, both with visual scaffolds, showed large improvements (64–74% correct posttraining, ~55% after 1 week) compared with controls. Crucially, Bayes factor (BF₀₁ = 5) provided evidence for lack of difference between conditions, leading the authors to conclude that training success arose from nested sets transfer and that this “finding supports the problem-representation transfer hypothesis rather than the format-representation shift hypothesis” (p. 7). This interpretation identifies an important source of success—abstracting relational structure—but the nested sets account does not specify how individuals detect, internalise, and generalise this structure. The study therefore demonstrates a pathway to successful reasoning consistent with nested sets accounts, while revealing that the mechanisms of learning remain underspecified.

Hoffrage et al. (2015) extended training research to more complex diagnostic tasks. In simple problems, accuracy was much higher with natural frequencies (~45%) than with probabilities (~7%), and brief natural-frequency instruction produced strong generalisation to complex tasks (73–81%). Rule-based and translation training also yielded nontrivial success (18–40%). The authors argued that it is largely sufficient to instruct people in using natural frequencies in the basic task in order to ensure a generalization to and solution of complex tasks and situated natural-frequency trees within Gigerenzer’s adaptive toolbox, noting that “the human mind is equipped with an adaptive toolbox containing simple heuristics that allow ‘fast-and-frugal’ decisions” (p. 10). Yet they also acknowledged important limits, stating that with many cues “the corresponding frequency tree would carry 2048 natural frequencies . . . and with 20 cues this number would be over 2 million” (p. 9). These admissions imply that no single representational tool is universally optimal. The study demonstrates that rule-based and translation strategies can also succeed, but ecological rationality does not explain how these alternative tools become learnable or deployable.

Talboy and Schneider (2017) showed that even very brief (<10 min) hybrid tutorials combining representational scaffolds with procedural guidance substantially improved performance using natural frequency formats only (about 70–72% of trained participants versus just under 55% of controls produced at least one correct answer) and reduced reliance on incorrect heuristics. They concluded that “people can learn to understand the complex interrelated information within Bayesian inference problems” (p. 382). Their explanation emphasised surface representational clarity (e.g., highlighting the distinction between sensitivity and predictive value) but did not integrate these findings into ecological rationality or nested sets accounts, nor did it provide a mechanism for how such hybrid instruction fosters generalisable Bayesian competence.

Starns et al. (2019) developed a “spatial bars” visualisation that maps Bayesian updating onto proportional bar lengths. Training markedly reduced deviation from normative odds and benefits persisted even without external aids. In some cases, accuracy matched explicit instruction in Bayes’ theorem, and classroom-based learners could “reconstruct the Bayes formula by analogy” (p. 12). The authors proposed that spatial comparisons make the logic of updating transparent, but their account remained descriptive and they acknowledged limitations for extreme probability values. Importantly, the success of a probability-based visualisation sits outside the predictions of ecological rationality and nested sets, suggesting that scaffolds that reduce cognitive load and expose joint and conditional relations can support learning independent of frequency input.

Kunzelmann et al. (2022) examined Bayesian reasoning in advanced medical students using double-tree and net-diagram visualisations that explicitly displayed the full joint and conditional structure of diagnostic problems. Unlike most laboratory studies, these visualisations already contained the relevant probabilities or frequencies at each node, so the task required participants to identify and interpret the correct posterior within a structured representation, rather than to perform arithmetic from scratch. Performance was high across all conditions: students solved 73% of probability-format problems in probability double-trees and 70% in probability net diagrams, compared with 80% in the corresponding natural-frequency versions. Thus, when relational structure is externalised and learners possess relevant domain knowledge, probability-format Bayesian reasoning can approach the same accuracy as natural-frequency formats. In the discussion, the authors emphasise that these unusually strong probability-format results reflect the learners’ prior conceptual preparation rather than any special property of frequency representations: “The high performance of medical students in the probability versions in our study could indicate that medical students—unlike students in other disciplines—are able to distinguish the relevant posterior from competing quantities” (Kunzelmann et al., 2022, p. 8).

Two recent studies in applied education also highlight the role of prior knowledge in Bayesian reasoning training. Büchter et al. (2022), piloted a complex-learning curriculum with medical and law students using natural frequencies, visualisations (double trees, unit squares), and a four-component instructional-design principle (van Merriënboer & Kirschner, 2007)—a framework for teaching complex skills through scaffolded practice on whole problems that integrates conceptual, procedural, and automatised knowledges. Medicine and law students improved substantially in performance and covariation tasks, though law students showed unexpectedly stronger baselines that went unanalysed. Following this, Steib et al. (2025) conducted a full pre–post follow-up trial (*N* = 515 law/medicine students), comparing four computer-based courses (double tree/natural frequencies, unit square/natural frequencies, natural frequencies only, probability tree) against a control; all improved performance short- and medium-term, but gains depended strongly on prior mathematical achievement, leading the authors to conclude that “the learning of Bayesian reasoning with the strategies used in our study seems to be affected by the *Matthew effect*” (p. 15). Double trees benefited learners across ability levels, whereas unit squares imposed high representational demands. These results show that successful reasoning depends jointly on instructional design and learners’ existing knowledge.

Finally, Kim et al. (2024) compared natural-frequency tree training with conditional-probability formula training in South Korean medical students. Both interventions produced high posttraining accuracy (~86%) and meaningful retention. Crucially, effects depended on prior experience: students without previous instruction benefited more from natural frequencies, whereas those already familiar with formula methods often performed equally well or better with formulas. The authors interpreted this pattern through the *Einstellung effect* (Luchins, 1942), observing that learners tend to persist with familiar strategies even when alternatives may be simpler.

Collectively, training studies demonstrate that successful Bayesian reasoning arises through multiple representational and instructional routes. Ecological rationality and nested sets each account for aspects of these gains, particularly those involving natural sampling or explicit set structures, but success in probability formats, the influence of motivation and prior knowledge, and the effectiveness of diverse scaffolds all suggest the need to broaden theoretical accounts to incorporate learning, structured knowledge, and the flexible use of multiple reasoning strategies.

### Section 2.2: Visual, cognitive, and task-based moderators of successful Bayesian reasoning

A substantial body of research demonstrates that success in Bayesian reasoning is shaped by visual, cognitive, and task-based moderators of successful Bayesian reasoning. We review them in this section.

#### Visual representations

Visual aids reliably improve Bayesian reasoning across both natural frequency and probability conditions. Meta-analytic evidence indicates increases from 4% to 26% in probability formats and from 24% to 47% when added to natural frequency problems (McDowell & Jacobs, 2017; see also Cui et al., 2023, for a review of the types of visual aids used). Ecological rationality accounts treat visualisations as supportive tools. Gigerenzer et al. (2007) suggest that external visual aids do part of the cognitive work by transforming complex numerical information into an easy-to-grasp format, assuming that diagrams assist reasoners in accessing the evolved frequency input. Brase (2009) situates pictorial aids within the ecological rationality framework, in which their effectiveness is explained by how closely they resemble naturally sampled frequencies rather than by their ability to display abstract set relations. In this view, pictorial representations are most helpful when they depict individuated events or objects, because such displays align with the hypothesised frequency-coding mechanisms of the mind.

However, empirical findings reveal that the effectiveness of visual aids is not solely reducible to individuated countability. Sirota et al. (2014) manipulated iconicity while holding the diagram type constant and found no evidence that iconicity or the higher realism of the icons improved reasoning (Study 1 BF₀₁ = 2.91; Study 2 BF₀₁ = 13.16). Improvements occurred only when visualisations clarified set–subset relations that were verbally obscured. This pattern aligns with nested sets interpretations: diagrams facilitate inference because they make hierarchical relations explicit. Additionally, Johnson-Laird and colleagues (1999) argue that diagrams facilitate reasoning by making the structure of the possible cases explicit rather than by approximating experienced frequencies. In their mental-model theory, reasoners construct representations of the situations described in a problem, where “a mental model is defined as a representation of a possibility” (Johnson-Laird et al., 1999, p. 66). Because diagrams externalise these models, they make the extensional relations among categories—what belongs to which set in which possibility—transparent, supporting correct inference without appeal to evolved frequency-coding mechanisms.

Further evidence for the scaffolding role of visual aids comes from Gigerenzer et al. (2021), who showed that natural frequencies presented as icon arrays facilitated Bayesian reasoning in second-grade and fourth-grade children, with most fourth graders arriving at the exact Bayesian solution. Notably, the same visual scaffold enabled most children diagnosed with dyscalculia to solve the problems exactly.

Lastly Stegmüller et al. (2024) examined joint probabilities (e.g., having breast cancer and receiving a positive test result) and showed that, unlike standard Bayesian inference, probability formats often outperformed natural frequencies when people had to judge joint events. Probabilities yielded higher accuracy than frequencies in three of five representations (Bayesian text, net diagrams, and 2 × 2 tables), because these formats already display joint probabilities directly, whereas frequencies require identifying and combining counts, adding cognitive steps and thus increasing error.

Visual aids therefore constrain the space of plausible explanations. Their effects cannot be reduced to natural frequency input alone, because noniconic and noncountable diagrams often perform just as well as realistic displays, and they cannot be reduced to set relations alone, because many visualisations that formally encode nested sets still fail to produce high accuracy. What visualisations reliably provide is a structured workspace in which relations among priors, likelihoods, and reference classes can be represented, inspected, and coordinated. They externalise key relations, reduce working-memory load, and stabilise the mental models that reasoners must construct in order to integrate multiple probabilistic components. From this perspective, visual aids do not supply a special representational format; they scaffold the deployment of inferential competence—allowing knowledgeable or trained reasoners to execute Bayesian operations more reliably, while leaving many novices unable to do so even when the structure is made visible.

#### Individual differences

Another source of variation in success arises from cognitive ability. Measures of numeracy, cognitive reflection, working memory, and visuospatial ability reliably predict who succeeds across both conditional probability and natural frequency formats. Chapman and Liu (2009), Johnson and Tubau (2013), Lesage et al. (2013), Sirota and Juanchich (2011), Sirota et al. (2014), Brase and Hill (2017) and Brase (2021b) all report robust associations between individual abilities and normative responding.

Nested sets proponents interpret these findings as evidence that Bayesian reasoning depends on domain-general analytic resources required to detect and manipulate hierarchical relations. Chapman and Liu (2009) showed that higher-numerate participants were substantially more accurate than lower-numerate participants across both formats, a pattern consistent with the view that identifying the relevant set–subset structure benefits from analytic processing. Johnson and Tubau (2013) found that numeracy moderated the benefits of linguistic simplification: low-numerate participants succeeded only when both the text and numerical structure were made maximally transparent, whereas high-numerate participants performed well even under more complex formulations. Lesage et al. (2013) reported that working memory and cognitive reflection predicted accuracy even in natural-frequency conditions, suggesting that recognising nested relations draws on analytic processing rather than arising automatically from format. Likewise, Sirota et al. (2014) showed that cognitive reflection facilitated construction of the correct reference class and inhibition of misleading alternatives, which they interpreted as evidence for analytic engagement in relational reasoning.

By contrast, ecological rationality accounts explain individual differences in terms of variability in constructing a frequency-compatible representation. Brase and Hill (2017) show that numeracy and visuospatial ability predict Bayesian reasoning accuracy. Brase (2021b, p. 236) interprets these findings within the ecological rationality framework, arguing that these abilities reflect how well individuals can “transfer the written and drawn Bayesian tasks into an ecologically more suitable representation” and are not predictors of alternative reasoning strategies but indicators of how well the frequency representation can be constructed.

Both interpretations capture elements of the findings but remain incomplete. High-ability individuals often succeed even in probability formats, where ecological rationality accounts assume that natural frequency-based mechanisms are not engaged; conversely, many low-numerate participants fail despite receiving natural frequencies, even though nested sets accounts propose that such formats make the relevant set relations more transparent. Moreover, when accuracy in conditional probability conditions approaches floor levels (close to 0%), individual-difference measures cease to discriminate among participants, suggesting that deeper mathematical knowledge, representational fluency, and prior experience may underpin successful reasoning in ways not captured by general measures of cognitive ability.

#### Task structure, probing, and processing demands

Other findings show that success depends strongly on comprehension, attentional guidance, and the number of operations required for solution.

##### Mental steps and compatibility

Ayal and Beyth-Marom (2014) identify task complexity as a key determinant of success, showing that accuracy declines as the number of operations increases and improves when data and question formats are aligned. As they summarised, “performance on base-rate-like problems depends directly on the complexity of the task . . . governed by two factors: the number of mental steps needed to reach the normative answer, and the compatibility between the data and the question” (p. 229).

##### Phrasing and text simplification

Johnson and Tubau (2013) show that improving the linguistic structure of Bayesian problems directly improves relational reasoning. By manipulating syntactic and semantic complexity while holding numerical information constant, they demonstrate that accuracy increases when the text makes the relations between sets easier to identify and integrate. Their conclusion that performance depends on the “non-numerical complexity of the problem’s text” (p. 38) indicates that clearer wording enhances the ability to recover the underlying nested set structure, not just to process the numbers. Ottley et al. (2016) arrive at the same conclusion from a different angle. By comparing multiple text-only versions of the same Bayesian problem, ranging from standard formulations to versions using probing, explicit framing, and narrative, they show that accuracy can increase from about 5% to over 40% without adding any visual aids. Their key finding is that wording alone can transform performance because it changes how easily participants can extract and organise the relevant relationships. Khan et al. (2018) provide the mechanism that links these results. Drawing on cognitive load theory and principles of instructional design (i.e., Mayer, 2014), they show that poorly structured text forces participants to expend cognitive resources on decoding and integration rather than on inference. When the same information is segmented, signalled, and made structurally explicit in text, performance improves dramatically, even without diagrams, because the inferential model becomes easier to construct.

##### Probing questions

Cosmides and Tooby (1996) argue that people can produce Bayesian-consistent answers when problems provide the right cues to trigger natural frequency-based representations. Their experiments show that reframing questions to refer to counts within a population rather than to single-event probabilities dramatically increases accuracy, because it directs attention to the appropriate reference class needed for inference. Girotto and Gonzalez (2001) reach a different but complementary conclusion. They demonstrate that performance improves not because of natural frequencies per se, but because certain question forms explicitly require participants to compute the two components of a ratio—the subset of interest and the larger set it belongs to. When questions are structured to prompt reasoners to identify relevant data, accuracy rises even when information is presented in conditional probabilities rather than natural frequencies. Their experiments show that set–subset relations become transparent when the question makes them explicit, eliminating base-rate neglect without any format change.

In more recent work on *reference dependence*, the tendency to anchor on salient but inappropriate reference classes, Talboy and Schneider (2017, 2022) found that virtually all reasoners who did not determine the correct response utilized values directly from the problem, such as the superordinate sample size. Misalignment between the problem structure and the question reduced accuracy, whereas repeating the correct reference class improved performance, demonstrating and suggesting that attentional salience often overrides representational facilitation. Similarly, Johnson and Tubau’s (2013, 2015, 2017) work on *relational reasoning* and question–problem misalignment shows that many errors in Bayesian reasoning arise because the relational roles of sets change between the presented data and the question (see also Tubau, 2022). When the question requires a new reference class (e.g., all positive tests rather than all infected cases), many reasoners fail to remap the same subsets into their new structural roles, producing errors such as calculating P(D|H) instead of the correct P(H|D).

### Section 2.3: Additional accounts for successful Bayesian reasoning

Beyond ecological rationality and nested sets accounts, a growing body of work has identified process-level mechanisms that determine whether Bayesian facilitation is realised. These approaches do not treat success as an automatic consequence of representational format, but as the outcome of how reasoners construct, monitor, and revise internal models of the problem. In particular, they show that causal understanding, metacognitive control, and strategic flexibility can enable or block Bayesian inference even when the same statistical information is provided. The following perspectives provide insights beyond ecological rationality and nested sets for why facilitative formats sometimes succeed, sometimes fail, and why performance varies so sharply across individuals.

#### Causal models

Krynski and Tenenbaum (2007) proposed that success increases when problems activate a plausible causal model. They argued that errors arise when reasoners construct causal explanations misaligned with the statistical structure; conversely, adding mechanistic explanations help guide the reasoner to the correct response. In their experiments, providing a causal account of false positives (e.g., benign cysts producing positive mammograms) raised normative responding from 16% to 42%. Hayes et al. (2016, 2018) later showed that causal information sometimes reduced error magnitude but did not guarantee correct solutions, with the authors concluding that causal facilitation redirects attention toward specific pieces of statistical information rather than ensuring full Bayesian integration. Together, these findings show that successful inference can emerge when causal context clarifies how the pieces of information relate, but these mechanisms operate independently of representational format.

#### Verbal comprehension, relational reasoning, and metacognition

Johnson and Tubau (2015) conceptualised Bayesian reasoning as a multi-stage process involving comprehension, construction of an internal representation, identification of the target relation, and computation. Crucially, success depends not only on representing nested sets, but on metacognitive control over how those representations are used. They argue that “reducing processing demands and/or increasing processing resources are two complementary means to the same end, a Bayesian response” (p. 15), highlighting that Bayesian performance reflects the ability to monitor, inhibit, and reassign relational roles when the question requires a different reference class. Later work shows that errors such as the hit-rate fallacy arise when automatic surface-level matches are not overridden, whereas correct responses require active realignment of focal and reference sets (Johnson & Tubau, 2017; Tubau, 2022). These accounts therefore locate Bayesian success not only in representational format, but in the executive regulation of structural mapping, providing a cognitive bridge between ecological rationality and nested sets accounts. Additionally, these authors emphasise verbal comprehension, treating Bayesian word problems as fundamentally verbal comprehension tasks: before any inferential process can begin, the problem must be understood well enough to construct an adequate problem representation (Johnson & Tubau, 2013, 2015). Sirota et al. (2024) extended this argument by demonstrating that wording varies systematically across statistical formats, confounding format comparisons, and that even reasoners capable of intuitive Bayesian inference from experience must be good readers to solve textbook problems. This account draws on established theories of text comprehension and mathematical word-problem solving, which describe how linguistic input is transformed into coherent problem representations (Kintsch, 1988; Kintsch & Greeno, 1985). When the text supports the identification of relevant referents and relations, reasoners are better able to map the problem onto its inferential structure; when it does not, performance is likely to suffer irrespective of the numerical format. In this sense, facilitation effects associated with visual or statistical representations can be understood as operating through their contribution to representation building, rather than as altering the inferential process itself.

#### Einstellung and cognitive rigidity

The Einstellung effect (Luchins, 1942) illustrates how entrenched strategies can block more efficient reasoning. Weber et al. (2018) observed that many participants, even when given natural frequencies, converted them back into probabilities, noting that “such an Einstellung toward calculating with probabilities instead of natural frequencies would take away all benefits that come with the frequency concept” (p. 4). This reveals that some failures arise from rigid application of familiar routines rather than representational difficulty. It also explains why success varies across learners: those who flexibly adopt new representations or strategies benefit more from facilitative formats. Kim et al. (2024) found that prior familiarity with formulas shaped which training method was most effective, concluding that learners tend to persist with the strategy they already know. Thus, the Einstellung perspective identifies success as contingent on flexibility and strategic adaptation based on prior learning.

### Section 2.4: The explanatory gap

The above observations reveal an explanatory gap: current theories illuminate why people succeed more in natural frequency formats than in conditional probability formats, but they do not fully explain how individuals develop the ability to reason successfully across tasks, formats, and levels of complexity. Across paradigms, success is achievable through multiple routes, yet current theories do not specify how the underlying inferential strategies are acquired, retained, generalised, or selected across tasks and representations.

## Section 3: Using Gigerenzer’s adaptive toolbox in Bayesian reasoning

This section argues that Gigerenzer’s adaptive toolbox provides an integrative framework for the success patterns reviewed in Section 2 by treating Bayesian reasoning as the deployment of multiple inferential tools. Applied in this way, the adaptive toolbox framework can accommodate training effects, individual differences, visual scaffolding, and expert performance within a unified explanatory framework. Examining how the adaptive toolbox has been applied within Bayesian reasoning further suggests opportunities for extending its scope to encompass a broader range of successful reasoning strategies. This section therefore clarifies the adaptive toolbox as a general framework, reviews its use in Bayesian reasoning, and identifies the explanatory questions that motivate the inclusion of the expertise-based account developed in Section 4.

### Section 3.1: The adaptive toolbox as a general framework

Gigerenzer’s (e.g., 2001, 2008) broader programme of ecological rationality proposes that the human mind possesses an adaptive toolbox containing heuristics—simple, structure-sensitive strategies that allow making judgements and decisions— rather than an optimisation or exhaustive computation mechanism. Gigerenzer proposes that the adaptive toolbox is a repertoire of cognitive strategies suited to different environmental structures for solving specific problems. Importantly, the toolbox is not a metaphor for general cognitive ability; it is conceptualised as a structured set of heuristics shaped by ecological fit. A heuristic is defined as “a strategy that ignores part of the information in order to make decisions more quickly, frugally, and with little loss in accuracy” (Gigerenzer & Gaissmaier, 2011, p. 454). Heuristics, from this view, are not arbitrary shortcuts, but rules of thumb composed of a search rule, a stopping rule, and a decision rule (Gigerenzer & Goldstein, 1996; Todd & Gigerenzer, 2000). These strategies are psychologically plausible because they rely on minimal computation and exploit statistical regularities in environments, allowing people to perform well under conditions of risk and limited resources.

An example is the recognition heuristic. When choosing between two options, individuals infer that the recognised option has the higher value on a criterion of interest, deliberately ignoring further information—explained by Goldstein and Gigerenzer (2002): “The recognition heuristic follows particularly simple rules. Search extends only to recognition information, not to recall. Search is stopped whenever one object is recognized, and the other is not; no further information is looked up about the recognized object. The simple decision rule is to choose the recognized object” (p. 88). This illustrates a more general point: the core idea of ecological rationality is that a heuristic is ecologically rational to the degree that it is tuned to the information structure in the environment. When environmental structure and cognitive strategy are matched, simple heuristics can outperform more complex, optimising models.

The adaptive toolbox framework has been applied successfully across a wide range of domains to explain systematic patterns of human and animal behaviour under uncertainty. In perception–action tasks, simple heuristics such as the gaze heuristic have been shown to account for how outfielders catch fly balls and how animals intercept moving prey, despite the absence of explicit trajectory calculations or optimisation (Gigerenzer, 2008; McLeod & Dienes, 1996). In economic and everyday decision-making, satisficing and one-reason decision strategies have been used to explain how individuals make effective choices under time pressure and limited information, often outperforming more complex optimisation models in ecologically realistic environments (Gigerenzer & Selten, 2001; Simon, 1956). In social and biological contexts, fast-and-frugal heuristics have been documented in mate choice, foraging, and risk-sensitive behaviour, where organisms rely on a small number of informative cues rather than integrating all available information (Hutchinson & Gigerenzer, 2011; Todd & Gigerenzer, 2000). Across these domains, the adaptive toolbox has provided a principled account of why multiple strategies coexist, how strategy success depends on environmental structure, and why learning and experience shape which tools are deployed in practice.

This broader adaptive toolbox perspective can also be applied to Bayesian reasoning, and prior work has already begun to move in this direction. In particular, frequency-based toolbox models of Bayesian reasoning (Hoffrage et al., 2015) and recent comparisons of single-process explanations with multi-strategy, toolbox-based models of Bayesian reasoning (Woike et al., 2023) illustrate how Bayesian performance can be understood in terms of strategy repertoires rather than a single inferential mechanism. The present review adopts this same toolbox orientation and seeks to extend it by integrating learning and expertise as central explanatory components of Bayesian reasoning.

### Section 3.2: Applications of the adaptive toolbox in Bayesian reasoning

Within Bayesian reasoning research, the adaptive toolbox framework has been most extensively developed through work on natural frequencies. Hoffrage et al. (2015) provide one of the most comprehensive articulations of this ecologically rational frequency-based toolbox approach to Bayesian inference. They describe a family of inferential tools designed to support accurate reasoning when information is presented in natural-frequency formats, including natural-frequency trees for both simple and complex Bayesian tasks, frequency grids, visual aids such as icon arrays, and instructional procedures for translating probability information into natural frequencies. Each of these tools is motivated by the idea that natural frequencies preserve the natural sampling structure of the problem and reduce the computational demands associated with conditional probabilities.

More recently, Woike et al. (2023) explicitly tested a toolbox approach by modelling participants’ responses as arising from a set of distinct inferential rules rather than from a single underlying process. Using data from more than 10,000 Bayesian inference responses, they identified a toolbox consisting of six rules applied to probability-format problems: five non-Bayesian heuristics—representativeness, base-rate only, false-alarm complement, likelihood subtraction, and joint occurrence—and Bayes’ rule itself. Responses were classified as reflecting a specific rule only when the numerical answer matched that rule’s prediction within a narrow tolerance (±0.1%), allowing precise attribution of strategy use.

To validate these classifications, Woike et al. (2023) conducted three additional experiments examining response times and self-reported information use. Participants classified as Bayes’ rule users overwhelmingly reported using all three relevant probabilistic quantities (base rate, hit rate, and false-alarm rate), whereas users of simpler heuristics reported relying on fewer pieces of information. Response-time analyses further aligned with theoretical expectations: responses classified as Bayes’ rule took longer on average than heuristic-based responses, consistent with differences in computational complexity. Overall, the toolbox model accounted for approximately 64% of responses to probability-format Bayesian problems and provided a better fit to the data than single-process models.

These applications of the adaptive toolbox framework can account for several Section 2 patterns: training effects reflect tool acquisition, visual scaffolding makes frequency-based tools accessible, and individual differences map onto variation in deployed rules. However, the adaptive toolbox framework can be further expanded to identify additional tools and explain other success phenomena. Specifically, high accuracy in probability formats without representational scaffolding, and expert performance across formats, suggest tools beyond those currently characterized in Bayesian reasoning research.

### Section 3.3: Evidence for a broader account

Identifying these additional tools requires examining what current applications emphasize. Hoffrage et al. (2015) explicitly acknowledge that “the mind may have other tools in the toolbox” (p. 14). However, successful Bayesian inference within this framework is typically supported by representational scaffolds that transform probabilistic information into natural frequency-like formats, rather than by heuristics that operate directly on probability representations. But no parallel heuristics are proposed for facilitating Bayesian inference when information is presented in probability formats. For example, in Hoffrage et al. (2015) authors discuss natural frequency-derived heuristics in comparison to models of such as logistic regression, Classification and Regression Trees CART, and Naïve Bayes (see Laskey & Martignon, 2014, for an explanation of these), these models are offered as statistical benchmarks rather than simple heuristics for reasoning with probabilities.

The strategy-based modelling developed by Woike et al. (2023) further clarifies both the strengths and the scope of current toolbox applications to Bayesian reasoning. Although Woike et al.’s validation approach demonstrates that participants classified as “Bayes’ rule users” accessed the correct numerical information—all three probabilistic values—it does not establish that they performed the specific multiplicative operations prescribed by Bayes’ theorem. Their method identifies who arrives at the Bayesian answer and which information they consulted, but not the precise algorithm or reasoning pathway used to combine that information. Participants could potentially draw on the same three pieces of information yet apply different computational procedures or rely on alternative heuristics that happen to converge on the normatively correct answer for particular problem structures.

This procedural distinction matters because alternative procedures may constitute distinct tools within the adaptive toolbox. A simple pedagogical analogy illustrates the point: A learner who has not yet mastered multiplication may struggle with 3 × 5 as an abstract operation but readily solve the same problem through repeated addition (5 + 5 + 5), because this procedure aligns with existing counting routines. Although the numerical outcome is identical, the cognitive operations involved differ fundamentally. Bayesian reasoning tasks may operate similarly: individuals who succeed need not be implementing Bayes’ theorem as a formal algorithm but may instead rely on acquired schemas for interpreting proportions, conditional relations, or causal structure that support alternative reasoning pathways.

This insight highlights a deeper question in how the field conceptualizes Bayesian competence. Unlike classic fast-and-frugal heuristics, valued because they are simple, ecologically valid, and usually accurate, the “non-Bayesian” heuristics that dominate Bayesian reasoning tasks systematically produce incorrect answers (see Gigerenzer & Hoffrage, 1995, for an account of non-Bayesian algorithms). In tasks where complete probabilistic information is stated, and a normatively correct answer exists; these rules do not represent adaptive shortcuts to accurate inference—they represent distinct paths to error. Natural frequencies solve this by allowing simple counting to align with normative Bayesian computation. This raises the question: If the adaptive toolbox contains multiple heuristics, why is Bayesian reasoning with probability formats effectively left with one viable tool—Bayes’ rule (rarely used correctly)? One potential answer is that a broader set of tools is needed to be identified, one that includes exploration of heuristics suited to probability formats to simplify complex or abstract statistical structures. More generally, the field has rarely examined the strategies of the minority who succeed reliably in probability formats, despite their theoretical importance for explaining competence. Understanding whether these successful reasoners employ genuine Bayesian computation, functionally equivalent alternatives, or task-specific schemas would significantly advance our theoretical account of probabilistic reasoning.

Taken together, these considerations suggest that existing toolbox applications capture important aspects of Bayesian reasoning while leaving open questions about the full range of strategies that can support accurate performance, especially among individuals who succeed reliably in probability formats without representational translation to natural frequencies. The adaptive toolbox provides the right integrative framework for understanding these diverse success patterns—but realizing its explanatory potential requires solving a fundamental problem that current applications have left largely implicit: how do individuals acquire the tools in the first place, and how do they select among them?

### Sectio 3.4: The problem of tool selection

A central challenge for any toolbox model is explaining how individuals identify the appropriate tool for a given Bayesian reasoning task. Ecological rationality proposes that performance depends on a match between strategy and environment. Gigerenzer et al. (2003) drew on the Selection–Optimization–Compensation (SOC) framework (Baltes, 1997) to illustrate how individuals may adapt their strategy use across changing demands: selecting from their repertoire, refining strategies through practice, and compensating for limitations by shifting to simpler heuristics when required.

Additionally, Gigerenzer’s broader adaptive-toolbox programme extends this picture by treating strategy choice as shaped by individual reinforcement learning and social learning, with formal models such as strategy-selection learning theory specifying how people come to favour some heuristics over others based on feedback (Gigerenzer, 2008; see also Rieskamp & Otto, 2006). Before a heuristic is applied, individuals typically make judgements about its applicability and its ecological rationality for the present environment. Empirical findings show some sensitivity to environmental structure, for example, greater use of the recognition heuristic when recognition validity is high, or increased reliance on take-the-best when cue-validity variability is large (Goldstein & Gigerenzer, 2002). However, this adaptivity can be limited. Once strategies become habitual, they tend to lose responsiveness to changes in task structure (see Bröder & Schiffer, 2006).

Applications of the adaptive toolbox framework to Bayesian reasoning accommodate key success phenomena reviewed in Section 2, including training effects, visual scaffolding, and individual differences. At the same time, these applications leave open a central explanatory question: how do individuals acquire, organise, and reliably select appropriate inferential tools across tasks and representational formats? Section 4 integrates the adaptive toolbox framework with insights from expertise research to attempt to specify these mechanisms—how inferential tools may be acquired through structured practice, how they become automatised through experience, and how domain-specific knowledge structures enable context-sensitive tool selection—thereby extending the adaptive toolbox framework to an account of how Bayesian competence is built.

## Section 4: Expert tools in the adaptive toolbox

This section proposes that expertise research supplies the mechanisms required by the adaptive toolbox to explain how inferential tools may be learned, automatised, organised, and selected through experience and feedback. An expertise account provides a promising way forward for integrating ecological rationality and nested sets account within an adaptive toolbox framework. Expertise research shows that high-level performance in complex tasks arises from the acquisition of structured knowledge representations, recognition of deep structural features, efficient pattern recognition, automatized cognitive routines, context-sensitive tool selection and innate abilities (Campitelli et al., 2024).

In extending Gigerenzer’s adaptive toolbox to Bayesian reasoning, we do not assume that people possess an innate repertoire of heuristics specifically tailored to textbook Bayesian word problems. Rather, textbook tasks can be viewed as controlled contexts in which more general inferential tools; such as, representing joint events, constructing marginal likelihoods, mapping reference classes, coordinating text-comprehension routines, and applying arithmetic procedures, can be acquired, refined, and automatised through learning and experience. From this perspective, performance on laboratory Bayesian reasoning tasks is informative because it reveals how learned tools are organised and deployed, not because the tasks themselves mirror everyday decision environments.

If Bayesian reasoning depends on selecting appropriate tools, then competence hinges not only on the knowledge structures that encompass the execution of skills or the expression of expert knowledge but also on dynamic knowledge structures that govern tool selection and execution. Thus, a broader and more complete account of Bayesian reasoning should expand the adaptive toolbox to include innate algorithms (e.g., the Bayesian algorithm that works with frequencies in Gigerenzer & Hoffrage, 1995), fast and frugal heuristics (e.g., the reasoning strategies presented in Woike et al., 2023), acquired knowledge structures that facilitate the creation of mental models of situations (e.g., tree diagrams, Euler circles, iconographs, mosaic plots, 2 × 2 tables; Johnson-Laird et al., 1999), domain-specific knowledge structures (e.g., the actual Bayes theorem), but also dynamic knowledge structures engaged in the selection of the appropriate tools for each situation. This proposal also attempts to address the tool selection challenge identified in Section 3. Expertise research suggests that reliable tool selection depends on acquired knowledge structures that enable recognition of deep problem features, automatized retrieval of appropriate strategies, and context-sensitive deployment of inferential tools. We develop this account in five steps: establishing the bridge from Gigerenzer’s intuition concept to expertise, outlining Simon’s expertise approach and paradigm, clarifying the expertise framework and addressing misconceptions, applying it to Bayesian reasoning, and articulating its explanatory advantages.

### Section 4.1: Intuition as expertise: An underdeveloped implication

Gigerenzer’s broader writings gesture toward an important but underdeveloped theme: intuition as a product of expertise. He has long described intuition as a form of ‘unconscious intelligence’ that allows individuals to make effective decisions without explicit deliberation (Gigerenzer, 2007). In Gigerenzer (2008), he further notes that “experimental studies indicate that people select different heuristics in different environments, intuitively evaluating their ecological rationality” (p. 25). This has been made explicit in Gigerenzer’s recent theoretical work where he defines intuition as “a feeling (1) based on long experience, (2) that appears quickly in one’s consciousness, and (3) whose underlying rationale is unconscious” (Gigerenzer, 2023, p. 3). Crucially, Gigerenzer emphasises that intuitions are domain-specific and depend on extensive training with feedback, noting that “the superior intuitions of experts require extensive training, with elite performance estimated at some 10,000 hours of deliberate practice” (Gigerenzer, 2023, pp. 3–4). This implies that heuristics become intuitive not because they are innate modules, but because they are learned, selected, and organised through experience with structured environments. Thus, intuition may not be considered a general cognitive ability, but the automatised expression of learned inferential structures within a domain.

This perspective provides a natural bridge from ecological rationality to an expertise framing. If successful reasoning depends on selecting and deploying appropriate heuristics, then competence must depend on the acquisition of the knowledge structures that make such selection reliable. In Gigerenzer’s own formulation, expert intuitions emerge because of experience, such that “options come to mind in the order of their validity” for skilled decision makers (Gigerenzer, 2023, p. 4). What feels like a ‘first hunch’ is therefore the output of a highly trained heuristic system, not a guess.

Viewing intuition (or, at least, some forms of intuition) as the gradual internalisation of expertise shifts the explanatory target. Intuitive reasoning reflects the automatisation of cognitive routines shaped by practice, guided feedback, and repeated exposure to structured problem environments. Understanding Bayesian reasoning, therefore, requires an account of how these tools are developed, how they are selected in context, and how prolonged experience transforms explicit strategies into intuitive, context-sensitive forms of mastery. The role of domain-specific knowledge in tool selection can already be seen in ecological rationality’s account of heuristic success in medicine.

Clinical decisions often succeed not because physicians compute probabilities, but because they rely on domain-specific knowledge structures: learned cue hierarchies, symptom patterns, and clinically meaningful predictors refined through training and experience (Marewski & Gigerenzer, 2012). Fast-and-frugal trees work well in part because they formalise these existing clinical cue structures, exploiting statistical regularities embedded in medical environments. Yet clinicians frequently struggle with probabilistic quantities such as false-positive rates and post-test probabilities, indicating that these heuristics capture clinical expertise rather than probabilistic expertise. This distinction matters for Bayesian reasoning: medical heuristics demonstrate how experiential knowledge supports effective decision-making, but they do not explain how individuals recognise the structure of probabilistic information or select strategies suited to inference under risk.

This represents a major theoretical opportunity: to complement ecological rationality with a systematic account of the expert knowledge structures that make accurate Bayesian inference possible. The next section provides an overview of expertise research, which forms the basis of what we refer to as the expertise approach.

### Section 4.2: The expertise approach and expert-novice experimental paradigm

To specify how domain-specific knowledge structures develop and support tool selection, this section draws on Herbert Simon’s expertise approach in reasoning and decision-making (see Campitelli & Gobet, 2010). We believe incorporating Simon’s expertise approach to Bayesian reasoning is opportune as Herbert Simon’s bounded rationality framework predates both Tversky and Kahneman’s heuristics-and-biases programme (represented in Bayesian reasoning by nested sets accounts) and Gigerenzer’s ecological rationality approach and provides a common conceptual backdrop for contemporary debates about human rationality.

Simon’s (1955, 1956) foundational work proposed that human cognition is adaptive within constraints of time, knowledge, and computational capacity. His studies of chess masters (Chase & Simon, 1973; Gobet & Simon, 1996) suggest that expert performance arises from experience-driven learning rather than innate cognitive superiority. Experts develop specialized memory structures through deliberate practice: initially “chunks” encoding familiar patterns (Chase & Simon, 1973), then more elaborate “templates” (Gobet & Simon, 1996) with flexible slots for context-specific information. Moreover, masters use a heuristic called “satisficing”, which involves setting a satisfactory goal, rather than an optimal goal, before starting evaluating options to decide which move to play. When the evaluation of an option (i.e., a move to play) guarantees the achievement of that goal, the thinking process stops and that option is chosen. This strategy allows masters to avoid analysing all possible options, which is impossible given the complexity of the search space.

Campitelli and colleagues (2024) argue that expert memory research challenges traditional fixed-capacity memory models by showing memory limits can be dramatically exceeded through expertise. Ericsson and Chase (1982) demonstrated university students could increase their digit span from 7 to more than 80 items by using retrieval structures, organizing digits through their running time expertise. This work inspired theories proposing that experts circumvent memory limitations by leveraging long-term knowledge structures (Ericsson & Kintsch, 1995) and templates (Gobet & Simon, 1996) rather than expanding capacity to optimise efficiency. Contemporary competitors in World memory championships exemplify this: they routinely memorize thousands of digits or dozens of card decks by encoding information through systematic knowledge structures, converting numbers into familiar images, people, or actions organized spatially (i.e., method of loci; Campitelli et al., 2024; Fenwick et al., 2024; Guida & Campitelli, 2019).

Campitelli (2015) has extended this framework by conceptualizing knowledge structures as the cognitive basis of expertise that can be applied to understanding the human mind more broadly. Knowledge structures encompass both nonslotted schemas (knowledge in the form of letters, words, sentences) and slotted schemas (structures with modifiable placeholders such as the templates proposed by Gobet and Simon, 1996; see Guida & Campitelli, 2019, for a full account of knowledge structures as slotted and nonslotted schemas)

### Section 4.3: Expert versus novices research paradigm

Expertise research not only provided rich theoretical contributions but also developed an empirical paradigm to study cognition. It simply consists of comparing a group of experts with a group of novices or, even better, to use participants along a wide spectrum of expertise from novices to experts and submit them to domain-specific and non-domain specific tasks (see Campitelli & Gobet, 2010). This approach complements the traditional learning paradigms in which participants are trained to achieve a level of expertise (e.g., Anderson, 1982; Logan, 1988). The learning paradigm allows researchers to heavily control confounding variables and observe the progress from novice to expert in a few hours, with expertise in this case being achieving automation in a specific task. However, this paradigm uses artificial tasks, and they do not capture types of expertise that require thousands of hours to achieve high levels of performance. The expertise paradigm allows to observe the performance of participants at very different levels of expertise, and it studies relevant types of expertise such as those in science, sports, arts, games and professions. A fruitful approach within this paradigm is process tracking methods such as think aloud protocols of experts while solving domain-specific problems (e.g., De Groot, 1978), eye-tracking to observe the aspects of stimuli experts pay attention to (Reingold et al., 2001) and brain imaging to map systems involved in cognitive processes.

### Section 4.4: Clarifying the expertise account

A common criticism to attempts to use expertise frameworks to illuminate theories and research of general cognition is that studying expert performance is irrelevant to understand the structure and dynamics of the untrained human mind. However, as Campitelli (2015) indicated, the untrained human mind does not exist. The only way of studying the untrained human mind is studying newborns, research that has provided important theoretical insights of innate capacities, but also has encountered obvious methodological problems (e.g., Oakes, 2017; Spelke & Kinzler, 2007; Xu & Garcia, 2008). When studying adults, attempts to block the knowledge of participants by excluding criteria (e.g., in Bayesian reasoning excluding expert mathematicians or statisticians), or observing the difference of performance between conditions are futile. Participants need to use their knowledge to at least read the instructions of the experiment, and, in the case of Bayesian reasoning, this knowledge is required to read the stimulus (i.e., the vignette with the problem to be solved). This is consistent with instructional design arguments from cognitive load theory, in which prior knowledge is central: “intrinsic cognitive load cannot be altered by instructional interventions because it is determined by the interaction between the nature of the materials being learned and the expertise of the learner” (van Merriënboer & Sweller, 2005, p. 150). Thus, ignoring the level of expertise of the participants, is missing an opportunity to see the relationship between the knowledge and skills of participants and performance in Bayesian reasoning. Research in Bayesian reasoning had incorporated the investigation of individual differences in numeracy, cognitive reflection, working memory and other measures, and that is a good starting point; studying experts increases the spectrum of knowledge and skills, providing additional information.

Another misconception may be self-inflicted by the field of expertise research. The most popular approach is the deliberate practice framework (Ericsson et al., 1993), which indicates that the level of expertise is only a function of the accumulated hours of deliberate practice, with no effect of innate individual differences whatsoever. This conception makes it difficult to relate the knowledge structures acquired by practice with those extant at birth as the outcome of an evolutionary process. However, that conception has been challenged, with expertise being explained by domain-specific practice, innate individual differences, sensitive periods, and many other factors (e.g., Campitelli & Gobet, 2011; Gobet & Campitelli, 2007; Hambrick et al., 2014). Thus, an expertise approach is well positioned to consider the contributions of both innate and acquired knowledge structures and cognitive processes.

Expertise relevant to Bayesian reasoning may not be unitary: statistical, mathematical, domain-specific, and task-specific expertise need not draw on the same tools or produce the same patterns of reasoning. Statistical and mathematical training may supply formal and representational tools, such as Bayes’ rule, likelihood-ratio procedures, and internalised frequency structures, whereas domain-specific expertise may instead support causal modelling and reference-class knowledge without conferring the formal computation, and task-specific practice may automatise the solution routine for a particular problem type. We take statistical and mathematical experts as a starting point, since their success across formats offers a clear test of which formal and representational tools support inference. The account is not restricted to this group, however, because an expertise approach addresses reasoning across the full range of skill, the same paradigm extends to professional domains in which Bayesian inference is consequential, such as clinical diagnosis and legal or forensic judgment, where domain-specific expertise may recruit a different profile of tools from the toolbox.

### Section 4.5: Incorporating the expertise approach into Bayesian reasoning with the adaptive toolbox

In Bayesian reasoning specifically, we propose that experts have not circumvented evolution or our biological brain structures but have enhanced application of mathematical reasoning through development of domain-specific knowledge. This would include:

1. Well-structured probability concepts and their interrelations,
2. Automated computational procedures for probability transformations, and
3. Schemas for mapping problem features onto solution strategies.

Some aspects of this expertise can be acquired by relatively short training interventions to people who already have the knowledge of mathematics acquired at school. Evidence of this is the dramatic improvements observed in training studies, where average performance moves from 0–10% to 64–78% success rates on probability-format problems following instruction (e.g., Sedlmeier & Gigerenzer, 2001). Additionally, several Bayesian reasoning studies indicate that samples drawn from highly educated or elite institutions, such as MIT, show higher mean accuracy and reduced base-rate neglect compared with other samples, even though performance is still not close to 100% accuracy (see Brase et al., 2006, for examples and commentary on this.; Kim et al., 2024; Krynski & Tenenbaum, 2007; McNair & Feeney, 2015).

When incorporating the expertise approach to Bayesian reasoning it is paramount to connect the learning of new knowledge structures with the innate knowledge structures. As Gigerenzer (2008) notes “Heuristics exploit evolved capacities. The term evolved does not refer to a skill shaped by nature or nurture alone. Rather, nature provides humans a capability, and extended practice turns it into a capacity” (p. 25). An expertise framework contributes to understand how this transformation occurs; specifying knowledge structures, automatization processes, and memory reorganization through which practice converts capacity into expert capability. For example, the reason why some untrained participants are successful at solving Bayesian reasoning problems in the probability format may be due to two reasons. First, the participants received instruction on how to solve those problems that may include the Baye’s theorem or step-by-step instructions on how to solve the problem. Those knowledge structures (i.e., the Bayes theorem and the instructions) are tools available in the adaptive toolbox. Second, and more likely, participants successfully put together a set of knowledge structures that were not acquired specifically to solve this type of problem, but that lead to a successful solution. These knowledge structures may include mathematical operations and aids such as tree diagrams and 2 × 2 tables, as well as knowledge of how to translate a text into a model (mathematical or visual) of the problem.

#### Section 4.5.1: Tools for success in the adaptive toolbox

Drawing on the evidence reviewed in Sections 2 and 3, we propose five hypothesised families of inferential tools that may populate the Bayesian reasoning toolbox. These are not intended as an exhaustive taxonomy but as plausible candidates to be empirically specified and tested through the expert-novice experimental paradigm.

*Formal tools* could include Bayes’ rule as a computational algorithm (Gigerenzer & Hoffrage, 1995; Woike et al., 2023), odds-based and likelihood-ratio calculations (Starns et al., 2021), and computational templates acquired through rule-based training (Sedlmeier & Gigerenzer, 2001; Kim et al., 2024). We propose that additional probability-based procedures, such as likelihood-ratio updating, may constitute distinct tools available to mathematically trained reasoners.

*Representational tools* could include natural frequency trees and grids (Hoffrage et al., 2015), 2 × 2 tables, double trees and net diagrams (Kunzelmann et al., 2022), icon arrays (Brase, 2009), and spatial bar plots (Starns et al., 2019). Critically, these need not be externally present to function as tools: with sufficient practice, reasoners may deploy internalised mental models of previously learned visual scaffolds (Johnson-Laird, 1994), applying their structural logic without external aids, a mechanism that may account for expert success in probability formats where no representational scaffolding is provided.

*Structural and relational tools* could include schemas for mapping priors, likelihoods, and reference classes onto problem structures (Hoffrage et al., 2015; Johnson & Tubau, 2015), strategies for constructing joint and marginal probabilities, nested set and partitive-structures (Barbey & Sloman, 2007; Sloman et al., 2003), and routines for identifying and remapping reference classes when the question requires a different referent than the data presents (Johnson & Tubau, 2017; Talboy & Schneider, 2022).

*Causal tools* could include causal models of diagnostic problem structure by clarifying the causal pathways underlying the statistical relations (Krynski & Tenenbaum, 2007). These causal models can be formalised as directed acyclic graphs (DAGs), representational structures that make probabilistic dependencies between hypotheses, evidence, and alternative causes explicit, directly supporting the decomposition of statistical information required for Bayesian inference (Pearl, 2000).

*Text-comprehension tools* could include problem-translation routines for segmenting complex sentences, identifying relevant referents, and using paraphrase or self-explanation to recover the intended set-subset structure (Johnson & Tubau, 2013; Khan et al., 2018). Critically, the adaptive toolbox framework treats these not as background prerequisites but as acquired, automatised tools in their own right: knowledge structures that experts bring to Bayesian word problems and that novices must develop through instruction and practice. Building on Johnson and Tubau’s (2015) and Sirota et al.’s (2024) characterisation of Bayesian problem-solving as a text comprehension task, this fourth family is grounded in established accounts of how reasoners map linguistic descriptions onto mathematical structures to form coherent problem representations (Hegarty et al., 1992; Kintsch, 1988; Kintsch & Greeno, 1985). Instructional design research demonstrates that reducing extraneous processing demands improves complex problem solving, while expertise develops through the acquisition of knowledge structures that support efficient performance (Mayer, 2008; Sweller et al., 2019; van Merriënboer & Sweller, 2005). In Bayesian reasoning, domain experience facilitates recognition of problem structure, reducing representational demands across formats.

Different combinations of tools from these families may underlie successful Bayesian reasoning across tasks and formats. What distinguishes experts from novices, on this account, is not possession of a single privileged tool but the breadth, organisation, and flexible deployment of their toolbox, and the reliability with which appropriate tools are selected across representational conditions. This also explains why individual difference measures predict performance among novices but are expected to become weaker predictors as expertise develops: as tools are automatised and selection becomes efficient, the cognitive demands these measures capture are progressively reduced.

#### Section 4.5.2: The expert–novice paradigm for Bayesian reasoning: Studying success rather than failure

We propose incorporating expert–novice experimental paradigms to explore what heuristics or tools mathematical experts use and whether their strategies for probabilistic reasoning can be applied to novices through training, representation, or visual aids. This proposal follows the logic of the adaptive toolbox programme itself. Many of the heuristics that populate Gigerenzer’s toolbox were not discovered by analysing laboratory errors but by studying skilled performance in natural environments (Gigerenzer, 2008). An example is the gaze heuristic in ball catching: outfielders do not calculate trajectories, velocities, or landing points. Instead, they fixate the ball and move so that the angle of gaze remains constant, a simple rule that reliably produces interception in a wide range of physical environments (Gigerenzer & Gaissmaier, 2011). This heuristic was identified precisely because researchers examined how experts successfully perform the task, rather than how novices fail.

The same methodological principle applies to Bayesian reasoning: to discover what tools make probabilistic inference work in practice, one must examine how mathematically trained individuals solve probability-format problems, on top of inferring cognitive mechanisms from the errors of untrained participants. Moreover, when individuals do arrive at correct probability-format answers, current research typically assumes they have applied Bayes’ theorem as normatively specified (i.e., Woike et al., 2023). This assumption, however, remains largely unexamined. There are multiple pathways to correct solutions that may not involve formal Bayesian computation—approximation strategies, heuristic shortcuts, or alternative inferential approaches that yield normatively correct responses through different cognitive mechanisms. Without direct investigation of how individuals who reliably succeed with probability problems actually reason through these tasks, we cannot determine which cognitive tools are being deployed. Understanding the diversity of successful reasoning strategies requires examining expert performance through process-tracing methods.

Across statistics education, a central premise is that analysing how expert statisticians think provides a powerful foundation for improving novice statistical literacy and reasoning. Research in this tradition routinely examines expert practice, how statisticians integrate context, interrogate data, model uncertainty, and coordinate the full investigative cycle, to inform instructional design and guide learners toward deeper, more integrated forms of reasoning (Wild & Pfannkuch, 1999). Expert-derived frameworks underpin major reform movements in statistics education, where expert cognition is treated as a normative benchmark for cultivating statistical reasoning and thinking in diverse learner populations (Garfield et al., 2015; Greenhouse & Seltman, 2018). These accounts converge on the idea that instruction should explicitly target the reasoning behaviours characteristic of experts (see also van Merriënboer & Sweller, 2005), rather than rote procedural fluency, which fits together with our proposal to study Bayesian reasoning through an expert–novice paradigm that takes expert performance as the instructional and theoretical benchmark. While principles from mathematics education (Büchter et al., 2022) and mathematical cognition (Johnson & Tubau, 2015) have been included in training design and problem representation, expert-novice comparisons have not been readily tested. If expert cognition is central to advancing novice understanding in statistics education, the same logic should apply to Bayesian reasoning: Systematically studying expert–novice differences in probabilistic inference could reveal the cognitive tools and representational strategies that underpin successful Bayesian reasoning, offering a more robust theoretical foundation for intervention than format-based manipulations alone.

#### Section 4.5.3: Addressing the tool selection problem

In expertise research, comparing expert chess players with lower-ranked or novice players is standard practice because both groups share the minimal domain knowledge needed to perform the task, understanding the pieces, legal moves, and basic strategies (Chase & Simon, 1973; De Groot, 1978). Bayesian reasoning research relies on an equivalent baseline: participants must comprehend written instructions and carry out simple arithmetic. We would not administer a Bayesian problem to individuals with no literacy or numeracy. The Bayesian reasoning field has, however, concentrated on nonexpert participants (i.e., Brase, 2014; Khan et al., 2015; Ottley et al., 2016; Sirota et al., 2014). This focus has been productive: Edwards’s (1982) programme, for instance, used nonexpert samples not merely to document failure but to model how people integrate priors with new evidence. Sampling the upper end of the expertise spectrum extends this tradition rather than correcting it, completing a continuum that expertise research examines in full, but that Bayesian reasoning research has sampled only at its lower ranges.

Using the expert–novice paradigm provides a means of addressing the tool selection problem identified in Section 3. By examining how mathematical experts recognize Bayesian problem structures and deploy appropriate strategies, we can identify the knowledge structures that enable reliable tool selection—the schemas for conditional relations, the automatized recognition of deep structural features, the organized repertoires of solution strategies. These are precisely the mechanisms that the adaptive toolbox framework presupposes but that representational accounts have not specified. Where Section 3 showed that Bayesian reasoning requires multiple tools, the expert–novice paradigm could reveal how those tools are learned, organized, and selected in practice—transforming the adaptive toolbox from a collection of strategies into a functional cognitive system with a specified developmental trajectory. We emphasise, however, that this proposal remains programmatic. The framework specifies candidate tools and identifies the expert-novice paradigm as a means of investigating their selection, but the mechanism by which reasoners select among available tools has yet to be specified and tested empirically. Characterising how selection operates is an open question that the research agenda outlined here is designed to address.

## Conclusion

We argued that while representational facilitation in Bayesian reasoning is an important finding, understanding how individuals achieve successful Bayesian reasoning across diverse conditions is also a critical explanatory challenge. Natural frequencies, visualisations, and other representational aids reliably improve performance, yet these effects do not fully explain why some individuals reason accurately in probability formats without scaffolding, why training can produce durable improvements, or why experts consistently outperform novices under identical task conditions. To address these findings, we proposed integrating Gigerenzer’s adaptive toolbox with an expertise framework. Within this account, Bayesian reasoning depends not on a single format-sensitive mechanism, but on the acquisition, organisation, and flexible deployment of inferential tools through learning, instruction, and experience. Ecological rationality explains why some tools are effective in particular environments, nested sets theory explains why making relational structure explicit facilitates inference, and expertise research provides a mechanism for understanding how these tools and representations are acquired, automatised, and selected across contexts. This framework also motivates a future research agenda focused on identifying the tools that support successful Bayesian reasoning. Studies comparing experts and novices, combined with process-tracing methods such as think-aloud protocols, eye-tracking, written justifications, and other measures of reasoning processes, offer a promising approach for uncovering how successful inference is achieved and how these skills can be taught. By shifting attention from error to expertise, Bayesian reasoning research may move beyond questions of representational facilitation toward a broader understanding of how people learn to reason effectively under uncertainty.

## Data Availability

Not applicable.

## Footnotes

1 Most empirical work on Bayesian reasoning uses textbook problems with explicitly specified probabilities, which technically fall under decision making under risk (Volz & Gigerenzer, 2012). However, these tasks are often described more broadly as involving uncertainty in the psychological literature. In the present review, we adopt the term ‘risk’ for such well-defined problems. At the same time, the adaptive toolbox and expertise framework we develop is motivated by broader accounts of human cognition and is intended to provide a basis for explaining reasoning in less structured environments in which probabilities may be incomplete, ambiguous, or unknown. Establishing the extent to which these principles generalise beyond risk-based tasks remains an open empirical question.

2 It is important to note that Gigerenzer’s ecological rationality account, while primarily framed in terms of ecological fit, also treats reasoning strategies as evolutionary adaptations—that is, the mind has evolved capabilities and uses cognitive tools designed to enhance ecological adaptiveness. Evolutionary psychologists such as Cosmides, Tooby, and Brase place relatively stronger emphasis on specifying these tools as specialized modules, but this should not be read as a strict contrast between “ecological” and “evolutionary” accounts; rather, the two approaches overlap while differing in emphasis.
